# Operating Mechanism and Molecular Dynamics of Pheromone-Binding Protein ASP1 as Influenced by pH

**DOI:** 10.1371/journal.pone.0110565

**Published:** 2014-10-22

**Authors:** Lei Han, Yong-Jun Zhang, Long Zhang, Xu Cui, Jinpu Yu, Ziding Zhang, Ming S. Liu

**Affiliations:** 1 Centre for Cancer Molecular Diagnosis, Tianjin Medical University Cancer Institute and Hospital, National Clinical Research Center for Cancer, Tianjin, China; 2 State Key Laboratory of Agrobiotechnology, College of Biological Sciences, China Agricultural University, Beijing, China; 3 State Key Laboratory for Biology of Plant Diseases and Insect Pests, Institute of Plant Protection, Chinese Academy of Agricultural Sciences, Beijing, China; 4 Key Lab for Biological Control of the Ministry of Agriculture, China Agricultural University, Beijing, China; 5 Beijing Computing Center, Beijing, China; 6 CSIRO - Computational Informatics & Digital Productivity Flagship, Private Bag 10, Clayton South, Australia; Bioinformatics Institute, Singapore

## Abstract

Odorant binding protein (OBP) is a vital component of the olfactory sensation system. It performs the specific role of ferrying odorant molecules to odorant receptors. OBP helps insects and types of animal to sense and transport stimuli molecules. However, the molecular details about how OBPs bind or release its odorant ligands are unclear. For some OBPs, the systems' pH level is reported to impact on the ligands' binding or unbinding capability. In this work we investigated the operating mechanism and molecular dynamics in bee antennal pheromone-binding protein ASP1 under varying pH conditions. We found that conformational flexibility is the key factor for regulating the interaction of ASP1 and its ligands, and the odorant binds to ASP1 at low pH conditions. Dynamics, once triggered by pH changes, play the key roles in coupling the global conformational changes with the odorant release. In ASP1, the C-terminus, the N-terminus, helix α2 and the region ranging from helices α4 to α5 form a cavity with a novel ‘entrance’ of binding. These are the major regions that respond to pH change and regulate the ligand release. Clearly there are processes of dynamics and hydrogen bond network propagation in ASP1 in response to pH stimuli. These findings lead to an understanding of the mechanism and dynamics of odorant-OBP interaction in OBP, and will benefit chemsensory-related biotech and agriculture research and development.

## Introduction

Olfactory sensation is an essential capability for insects and mammals, enabling them to detect stimuli in the surroundings for prey, survival and reproduction [1], [2]. In the chemical-to-sensation process, odorant binding proteins (OBPs) ferry small, primarily hydrophobic odorant and/or pheromone molecules through sensillar lymph to olfactory receptors (ORs), triggering a cascade of chemosensory events which lead to activate sensory neurons [3], [4]. Signaling chemical molecule perception is particularly vital for many insects, such as social insect like honey bees, where the queen groups and controls the individual behaviors using sophisticated pheromone communication. Many studies have attempted to determine the key residues for OBP ligand recognition [5], binding and releasing [6]–[10]; however, the OBP's roles in delivering odorants has caused extensive debate [4], [11]. Therefore, the mechanism and dynamic pathways on how OBP binds and releases pheromones *in vivo* need to be explored at molecular level. An atom-level dynamics understanding of OBPs' binding and unbinding of odorant ligands, especially how pH affects the interactions between OBPs and their ligands, will help us understand the operating mechanism and functions of OBPs, ORs and the chemosensory system. This will assist with disease prevention, pest-control [12], food processes and agricultural technologies [1], [12].

**Figure 1:**
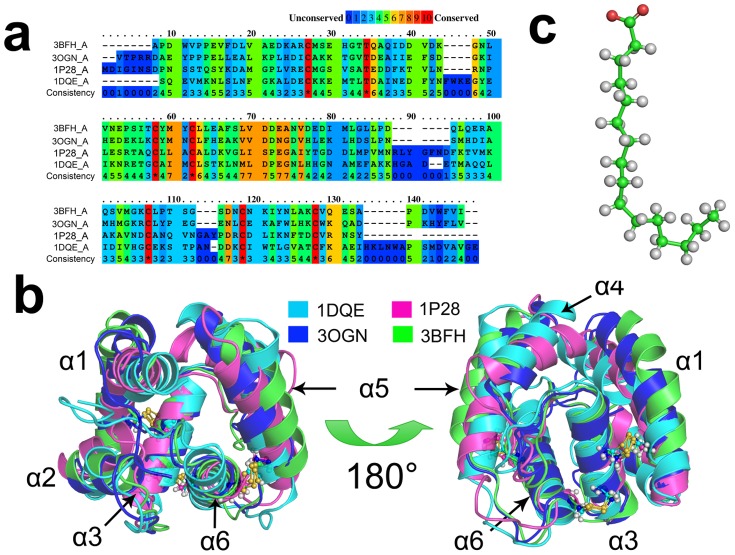
OBP sequences and structures. (A) The multi-sequence alignment for OBPs, 3BFH (PBP from honeybee *Apis mellifera*), 3OGN (OBP from the malaria mosquito *Anopheles gambia*), 1P28 (PBP from the cockroach *Leucophaea maderae*) and 1DQE (PBP from the silkmoth *Bombyx mori*). They represented the OBPs with different length of chain. The main difference of these OBPs is the length of C-terminal loop: 1DQE has a long *C*-ter, 1P28 has no *C*-ter while 3BFH and 3OGN have a mediate long *C*-ter. (B) The structure alignment for the above OBPs. Though they have low sequence similarity, they share almost identical tertiary structures, which imply that they share the same operating mechanism and dynamics. Three conserved disulfide bonds are shown as ball-and-stick models in yellow color. (C) The structure of palmitic acid, a typical odorant ligand.

The initial steps of chemo-sensing in honey bees involve the pheromone binding proteins (PBPs, one sub-type of OBP) binding to pheromone molecules, and carrying these ligands to ORs so as to activate ORs [3]. To date the accepted mechanism, as revealed by the crystal structures [7], [13]–[15] (see Fig.1), is that the process of OBP binding and releasing pheromone is to some degree pH-dependent. The same PBP and their ligands can be crystallized either in *apo* (ligand-free) or *holo* (ligand-bound) states subject to varying pH. Meanwhile, in an aqueous environment, there are different conformational states of the same protein at different pH [14]. In addition to honey bees, BmorPBP1 (PBP from Bombyx mori) structures show that the *C*-terminal loop is an important region in the presence of changing pH conditions [13], [14]. When BmorPBP1 is exposed to low pH condition (e.g. pH = 4.5), the *C*-terminal loop forms a new helix towards the binding cavity and pushes the odorant ligand out of the cavity. Conversely, at neutral pH condition (e.g. pH = 6.5), the unstructured *C*-terminus (*C*-ter) extends into the solvent and opens the binding cavity to host the ligand. Similar to BmorPBP1, ApolPBP (PBP from the giant silk moth *Antheraea polyphemus*) and AtraPBP1 (PBP from the navel orange worm *Amyelois transitella*) have the same long and unstructured *C*-ter as BmorPBP1, sharing a similar same mechanism in response to pH changes [16]–[18]. Unlike BmorPBP1, ASP1 (PBP from honeybee *Apis mellifera*) [19], AgamOBP1 (OBP from the malaria mosquito *Anopheles gambia*) [20], AaegOBP1 (OBP from the yellow fever mosquito *Aedes aegypti*) [21] and CquiOBP1 (OBP from southern house mosquito *Culex quinquefasciatus*) [9] do not contain a long loop at their *C*-termini, but their short loop could also fold into the binding cavity, occupy the binding site and disrupt the ligand's entry.

Earlier structural biology investigations [7], [13]–[17] have mainly focused on the functionality of the *C*-ter loop and key residues such as Asp35 in response to mutation and pH changes. However, they neglected the detailed and complete dynamics pathways on how OBP protein binds and unbinds its odorants, particularly at different conditions of pH. Recently, a few molecular modellings attempted to tackle the OBP- odorant dynamics and interactions [22]–[27]. These works encourage further investigation due to the still missing mechanisms, the lack of dynamics details and considerable uncertainty about the structure-function rationale. In this work, we chose honeybee *Apis mellifera* ASP1 as a model system and undertook long-time all-atom molecular simulations in order to elucidate the molecular mechanism and dynamics interaction of OBPs and its odorant ligands. ASP1, in significant contrast to many other OBPs, was reported to bind its ligands at lower pH condition while releasing them at neutral or high pH [7]. At the same time, ASP1 is structurally and genetically aligned well with many other OBPs (Fig. 1a & 1b). In our work, we focus on how pH affects the interation of OBP with its ligands and the mechanisms of OBP releasing ligand at a favorable pH condition. Through quantitative analysis of the global, local conformational changes and other dynamic properties of *apo*- and *holo*-ASP1s at pH 4.5 or pH 7.0, we try to illustrate a complete dynamics picture of molecular process how pH affects odorant release. We examined the dynamics contribution, not only of *C*-ter but also of *N*-ter, helix α2 and the region ranging from helices α4 to α5 (which form the entrance and core of the binding cavity), as well as its intrinsic disulfide bonds and hydrogen bond network.

## Results and Discussion

### Flexibility and fluctuation of the ASP1 structure

The global conformational changes in ASP1 with ligand (the *holo* state) and without ligand (the *apo* state) are depicted in Figure S2.1 in **Supporting Information S2**. The *holo* states are shown to have lower RMSD and RMSF than the *Apo* states (see Figure S2.1a & S2.1c in **Supporting Information S2**). Compared with the *apo* forms, the weaker conformation flexibility for the *holo* forms of ASP1 indicates that the ligand located in the hydrophobic cavity helps stabilize the overall structure of ASP1 and keep it dynamically tighter. The less flexible *holo* structures can help ASP1 carry the hydrophobic odorant molecules to the odorant receptor. The *apo* states of ASP1 at pH 4.5 condition appear to be more dynamic than the *holo* states (see Figure S2.1c in **Supporting Information S2**). This implies that the low pH condition provides the needed environment and dynamic condition for ligands to bind onto ASP1.

pH does have an effect on the flexibility of ASP1. As presented in Figure S2.1b, S2.1d & S2.1e in **Supporting Information S2**, in the *holo* state, RMSD and RMSF of ASP1 at pH 4.5 condition are definitely lower than the systems at pH 7.0 condition. The average values of RMSD (of 200 ns MD triplicates) for ‘*holo*-3BFH’ at low pH 4.5 and neutral pH 7.0 conditions are 1.56 and 2.02 Å, respectively, with standard deviation 0.15 and 0.29 Å. We found the same behavior in the ‘*holo*-2H8V’ state (see Figure S2.1a in **Supporting Information S2**): When set at pH 4.5 condition, its RMSD is about 1.37 Å with standard deviation 0.14 Å. At pH 7.0, RMSD fluctuates around 1.78 Å with a standard deviation of 0.33 Å. These observations indicate that the ASP1 structure at pH 7.0 undergoes greater conformational change and higher fluctuation during dynamics runs, and it is unfavorable for ligand docking to the cavity under pH 7.0.

**Figure 2:**
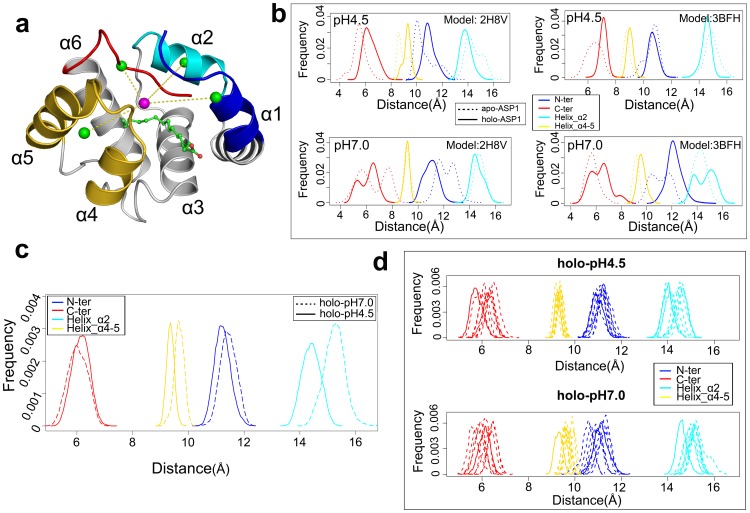
OBP binding cavity and its dynamics. (A) The ‘entrance’: four key components of the ‘entrance’ are drawn in different colors, with the center of the cavity represented in pink ball and the center of each key component depicted as green ball. The protein is presented by solid ribbons and the ligand molecule is in a ball-and-stick model. (B) The relative distribution of distance from the centers of each ‘entrance’ component to the cavity center for *apo*- and *holo*- states at same pH condition. (C) The relative distribution of distance between each entrance component and the entrance center for *holo*-state simulated at low pH 4.5 (solid lines) and neutral pH 7.0 (dash lines) conditions. (D) Time evolution of the relative distribution (with 20 ns interval) of each ‘entrance’ component to the center of cavity for *holo*-state at pH 4.5 (top) and pH 7.0 (below) conditions.

The *C*-terminal loop was believed to be important for odorant binding and releasing [13], [14]. For example in BmorPBP1, a pheromone-binding protein from the silk moth *Bombyx mori*, the transition from *holo*- to *apo*- BmorPBP1 comes when the *C*-terminal loop occupies the binding cavity in *apo* state [13]. A key question is whether the *C*-terminal loop folds into the binding cavity when the odorant ligand is released from the *holo*-ASP1, or it falls out of the cavity when the ligand is docked into the *apo*-ASP1? To explore these scenarios, we carefully looked at the trajectories simulated at pH 4.5 for the *apo* and *holo* states. Putting aside *N*-ter (a.a. 3 to 14) and *C*-ter (a.a. 111 to 119), as shown in Figure S2.1c in **Supporting Information S2**, the main fluctuating regions are the down-stream loop of α2 (a.a. 28 to 35), the loop between the helices of α3 and α4, and the region from α4 to α5 (a.a. 67 to 89). These fluctuating regions form the binding cavity, with the dynamic parts acting as a kind of ‘entrance’ of the cavity (see Fig. 2a, namely the ‘entrance’ formed by *N*-ter, *C*-ter and helix α2 and helices α4 and α5), which works to attract or eject the odorant ligands into/out of the cavity.

Given that RMSF only represents the flexibility residue by residue, we need to trace the forces of motion and other dynamic movement of the cavity during ASP1 actions. For this purpose, we monitored how the distances between the center of the cavity and four key cavity ‘entrance’ components change over the full trajectories. In this process, the centers of the total cavity (C_total_) and each component of the cavity ‘entrance’ (C_each_) were calculated using Eq. (2), then distances between C_total_ and C_each_ were measured as demonstrated in Fig. 2a. Fig. 2b shows that, except for helix α2, the other three components of the cavity entrance moved inwards the cavity in a coupling way and the binding cavity can contract without a ligand. This demonstrates again that *C*-ter is not the only region affected by the ligand, but the other parts of the whole cavity will respond to the ligand binding/releasing in a cooperative manner.

### ASP1's fluctuations response to the pH stimuli while the protonated residues do not directly interact with cavity/ligands

**Figure 3:**
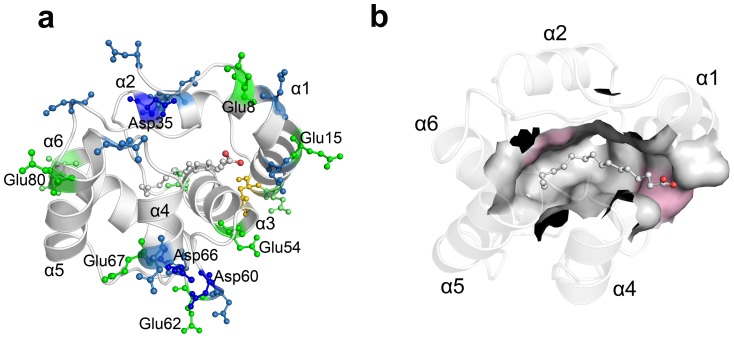
Distribution of ionizable residues versus the cavity. (A) Ionizable residues are highlighted by ball-and-stick model with different colors, aspartic acid (blue), glutamic acid (green) and histidine (gold); (B) The cavity residues, 5 Å from ligand, are shown as surface model. The hydrophobic residues are shown with white and the pink represented with aromatic residues. The ligand bound into the binding cavity is drawn in a ball-and-stick model.

Previous investigation argued that the pH condition might trigger the release of the odorant ligand [11], [14], [17]. For ASP1, the ligand could settle in the cavity at the low pH condition (e.g. pH = 4.5) and unbind from the cavity at the neutral pH condition [7]. The different pH conditions will of course induce a different protonation state of the ionizable residues (as described in the Methods section). The structural studies implied that the effect of pH is employed indirectly by Asp35 to bend or unbend *C*-ter against the cavity. Nonetheless, it is unclear how different pH conditions (with responsible residues protonated) trigger the OBP/PBP cavity to bind or unbind the ligand, or even further ferry odorant molecules through lymph to activate the odorant receptors. It would be straightforward to explain the pH effect if the protonated residues of ASP1 are located in the binding cavity and directly involved in the cavity-ligand interaction. However, as shown in Fig. 3a, due to the location and distribution of protonated residues in ASP1, the protonated residues are not able to directly mediate the interaction of ASP1 and its ligands. The residues which are around 5 Å of the ligand are mainly hydrophobic and aromatic residues and the ionizable residues are distanced from the binding cavity (Fig. 3b). Then how did the ionizable residues mediate the interaction between ASP1 and its ligands at different pH levels? The only answer lies in that the change of conformational flexibility and fluctuation of ASP1, induced by pH changes, will propagate and induce the dynamic changes to the cavity for binding or releasing ligands.

### ASP1 favors the low pH condition for binding the ligand

**Figure 4:**
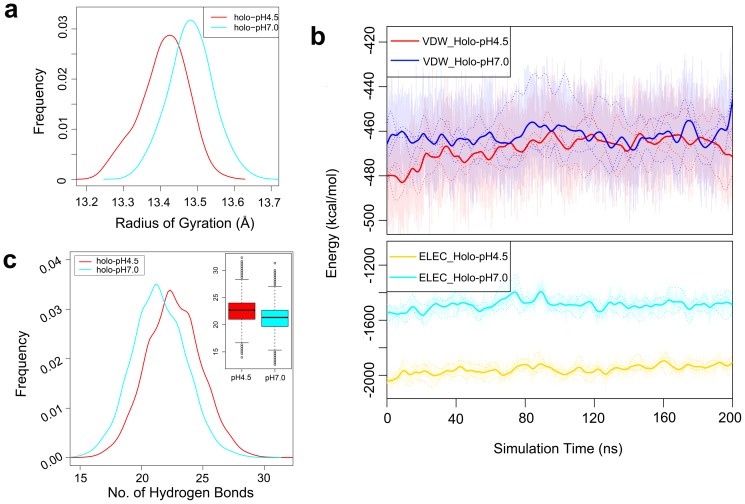
The radius of gyration *R*_g_ (A) and hydrogen bonds (C) distribution of *holo*- state simulated at pH 4.5 (solid red lines) and pH 7.0 (solid cyan lines) conditions. (B) The time evolution of van der Waals (top) and electrostatic (below) energy of *holo*- state simulated at pH 4.5 and pH 7.0 conditions, respectively. The dotted lines indicate individual trajectories of the three replicates.

Radius of gyration (*R*_g_) is a dynamic feature representing the structural compactness of protein [28]. As depicted in Fig. 4a, ASP1 at pH 4.5 has lower value of *R*_g_ than at pH 7.0 condition. The higher *R*_g_ value at pH 7.0 implies that the ASP1 structure becomes more dynamic at this pH condition, which is not favorable for keeping the ligand bound to the cavity. As also illustrated in Figure S2.1d in **Supporting Information S2**, the RMSF of ASP1 at pH 7.0 is larger than the case of at pH 4.5. The significant fluctuating regions, in addition to *N*-ter and *C*-ter, are α2 and the segment from α4 to α5. As discussed previously, these regions form an entrance and channel path for ASP1's cavity. The higher flexibility of these regions will make the cavity tend to unbind the ligand, instead of binding it.

From the above evidence, we conclude that the changes of global flexibility and fluctuation propagation as induced by pH changes define the operation mode of ASP1 and its cavity-ligand interactions. To test this hypothesis, we measured the time evolution of the cavity entrance's orientation and shape at different pH conditions. The details are shown in Fig. 2c. At pH 7.0, *N*-ter, helix α2 and helices α4and α5 are far away from the center of cavity while *C*-ter is close to the center of the entrance, in contrast to their position at pH 4.5. These give us a quantitative picture of how ASP1 releases its ligand out of the cavity. During the ligand releasing, *N*-ter, helix α2 and helices α4 to α5 will open up, facilitating the ligand's unbinding. At the same time, *C*-ter folds into the center and occupies the binding cavity, while helping to unbind the ligand from the cavity. This mechanism enforces the previous finding that *C*-ter plays a main role in responding to the change of pH condition [13], [14]. Generally, the pH changes should trigger a collaborative motion of not only *C*-ter, but also N-ter, helices α2, α4 and α5, and the coordination between *C*-ter and other cavity parts is vital condition.

Fig. 2c also shows that there is more than one peak for α2 and *C*-ter at pH 7.0 condition. This suggests that the ligand releasing is a dynamics propagating process. In other words, at pH 7.0 condition the cavity is more flexible, so as to transit in a series of conformation population (e.g. from opening to closing, and vice versa). The ligand is released from the binding pocket with certain higher probability. This assumption is confirmed by the conformation evolution as in Fig. 2d. In Fig. 2d, the total simulation time was divided into ten segments with the intervals of 20 ns. At pH 4.5, the cavity is very stable and dynamically constrained. But for ASP1 at pH 7.0, the four key components of the cavity, especially *C*-ter and helix α2, become very dynamic and undergo conformational transition. The higher flexibility of these components opens up the cavity (both volume and entrance) and shifts the dynamics probability towards the ligand releasing. At the same time, the opening of the cavity will reduce the interaction between ASP1 and its hydrophobic ligands. This means that the ligand will move more freely in favour of releasing.

ASP1, like most of OBPs, has three disulfide bonds in the core of the tertiary structure (Fig. 1b). Figure S2.1 in **Supporting Information S2** shows the regions near the disulfide bonds have low flexibility in comparison with other regions, either at pH 4.5 or at pH 7.0 condition. In contrast to the flexible cavity entrance, the disulfide bonds form a unique geometry plane (Figure S2.2a in **Supporting Information S2**), consisting of supra scaffolds, plays the role to stabilize the whole ASP1 structure. Due to the special role of these disulfide bonds, the fluctuation of geometry plane of three disulfide bonds reflect the exact motion of different regions in ASP1. As depicted in Figure S2.2b in **Supporting Information S2**, at pH 7.0 condition, the geometry plane of disulfide bonds is more dynamic (with larger fluctuation of planar angle) than at pH 4.5. This indicates that pH 7.0 is a condition for larger flexibility and will have the cavity opening for ligand releasing.

To quantitatively assess the binding energy of ligand to ASP1 as impacted by different pH conditions, we calculated by free energy perturbation (FEP) the binding energy difference at pH 4.5 and pH 7.0 condition (see Eq. (1)). The measured energy difference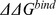 (for binding at pH 4.5 versus at pH 7.0) is −3.47 kcal/mol, see Figure S2.3 in **Supporting Information S2**. This binding energy difference clearly indicates that the ligand prefers binding ASP1 at low pH condition pH 4.5, rather than pH 7.0. Meanwhile, the relative small binding energy difference indicates that the interaction between ASP1 and its ligand is likely non-specific.

### Hydrogen bond network does matter: How the pH changes apply its influence

Through the above analysis we learn that the global flexibility and fluctuation play key roles in regulating ligand binding/releasing in ASP1. To further explore this mechanism, we considered three key non-bonded inter- and intra- molecular forces: the electrostatic, van der Waals and hydrogen bonds interactions at different pH conditions. In term of van der Waals energy, the ASP1-ligand complex at pH 4.5 is slightly lower than the counterpart at pH 7.0. However, the electrostatic energy of the ASP1-ligand (*holo* state) complex at pH 4.5 show much lower values than at pH 7.0 (Fig. 4b), indicating that the complex prefers the pH 4.5 condition. Hydrogen bond (H-bond) is another key factor reflecting the protein dynamics [29]. Fig. 4c shows that ASP1 possesses more H-bonds at pH 4.5 than at pH 7.0.

**Figure 5:**
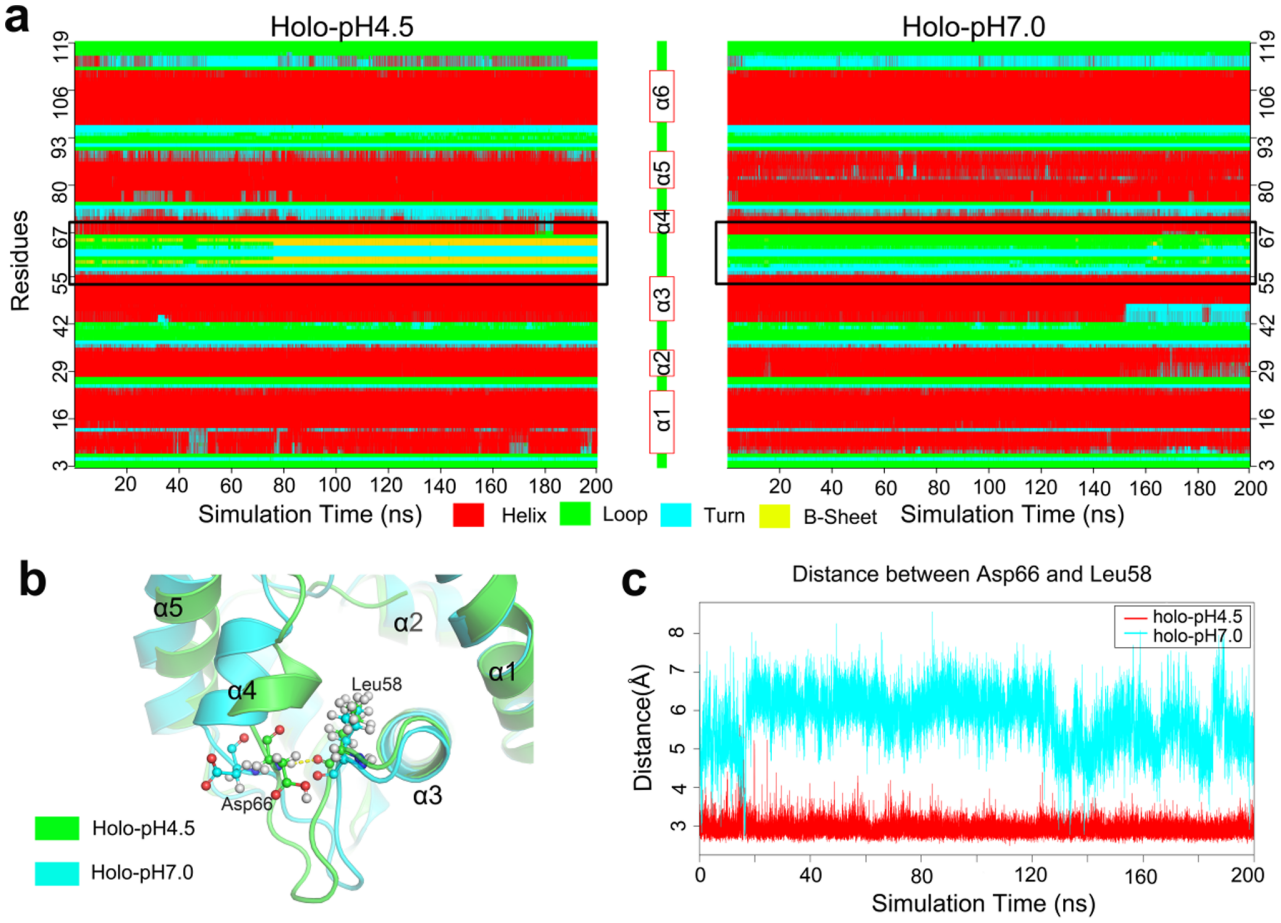
The time evolution of secondary structure and *H*-bond of *holo*-state simulated at pH 4.5 and pH 7.0 conditions. (A) The segment from Leu58 to Asp66 forms a beta sheet at pH 4.5 but always in a loop state at pH 7.0 condition. (B) A snapshot of the region from Leu58 to Asp66 of *holo* ASP1 at pH 4.5 (green) and pH 7.0 (cyan) conditions, respectively. Leu58 and Asp66 are highlighted in ball-and-stick, with its H-bond drawn as dotted line. (C) The hydrogen bond distances between Leu58 and Asp66 are depicted for pH 4.5 (red) and pH 7.0 (cyan) simulation conditions.

According to secondary structure analysis (with DSSP algorithm [30]), a beta sheet is formed between residues Leu58 and Asp66 at pH 4.5 condition, but this region is maintained as a unstructured loop at pH 7.0 (Fig. 5a). The H-bond between Leu58 and Asp66 is essential for the formation of the beta sheet. For example, at pH 4.5, Asp66 donates an H-bond to Leu58 over half of the trajectory time (Table 1 & Fig. 5c). However, there is no H-bond formation at pH 7.0. Structurally, the segment ranging from Leu58 to Asp66 connects α3 and α4 (Fig. 5b) and Leu58 to Asp66 are located at the two ends of this region. This specific H-bond can lock the beta sheet motif and thus maintain the rigidity of α4 and its neighboring region. As we know, α4 and its neighbor region are key components of the entrance of the binding cavity, so stabilizing this region can keep the cavity in the closed state for holding the ligand. At pH 7.0, the unprotonated Asp66 moves away from Leu58 and its chain stretches to the opposite direction to the configuration at pH 4.5 (Fig. 5b). With the loss of this H-bond ‘lock’, α4 and its neighbor part (as well as the cavity ‘entrance’), are destabilized and ligand unbinding will be induced.

**Table 1:** Hydrogen bonds occupancy in ASP1 at different pH conditions.

Hydrogen bond type	Occupancy (%)	
	Holo-pH 4.5	Holo-pH 7.0
N@Asp66: O@Leu58	50.58	0.31
OD2@Asp35: OT1@Ile119	19.31	∼0.00
OD2@Asp35: OT2@Ile119	18.23	∼0.00
NZ@Lys17: OT1@Ile119	22.38	17.07
NZ@Lys17:OT2@Ile119	17.29	13.82
NE1@Trp4: O@Val118	8.43	∼0.00
OH@Tyr48: OT1@Ile119	12.17	2.92
NE1@Trp116: O@Val34	16.65	0.96

**Figure 6:**
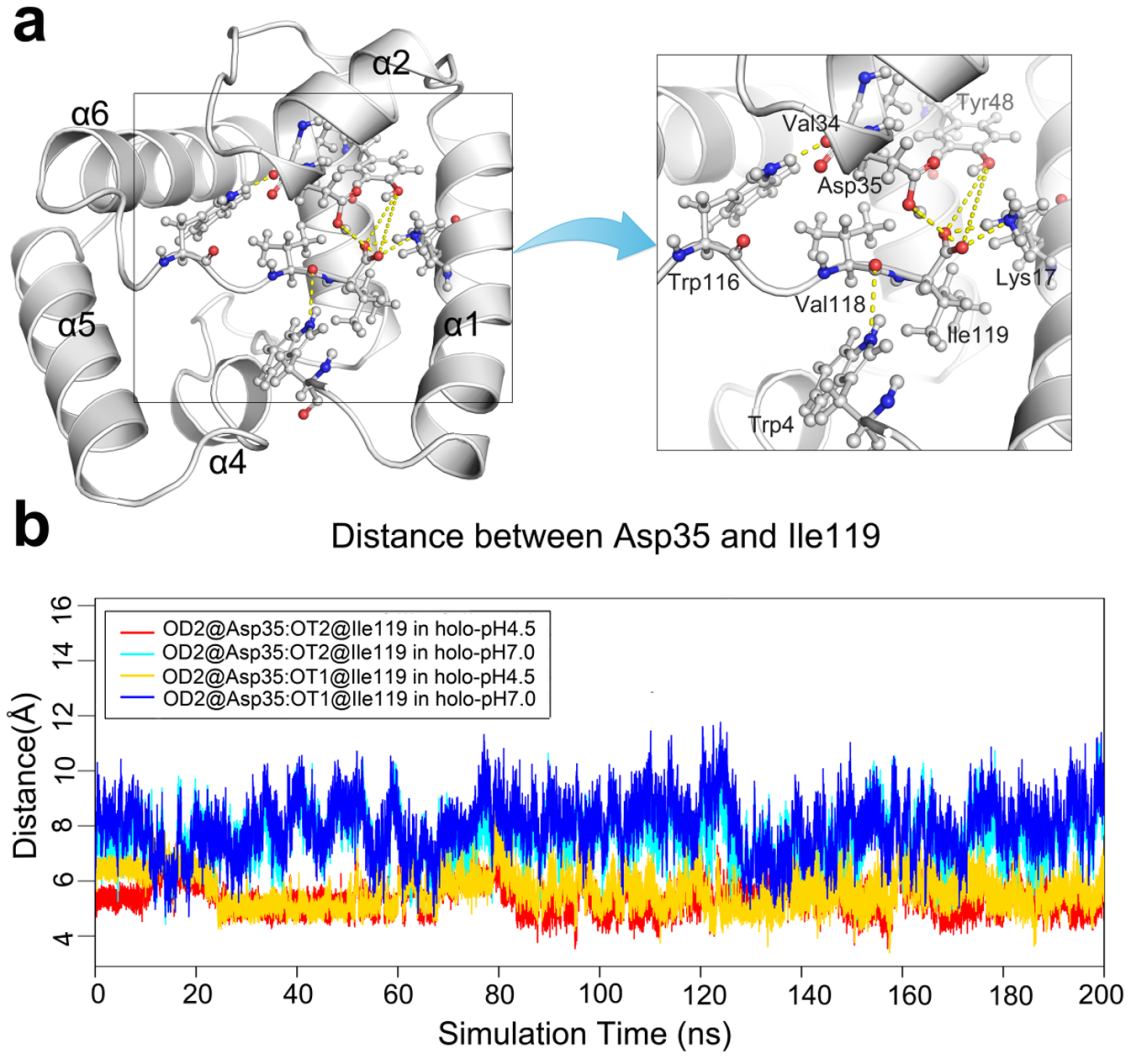
Dynamics of *H*-bond network. (A) Hydrogen bonds network formed by residues Trp4, Lys17, Val34, Asp35, Try48, Trp116, Val118 and Ile119. The residues participating in this hydrogen bond network are showed in the ball-and-stick models. (B) A time evolution of the bond distance between Asp35 and Ile119.

In addition to the Leu58-Asp66 H-bond, other H-bonds are formed by residues Trp4, Lys17, Val34, Asp35, Try48, Trp116, Val118 and Ile119, which connect *N*-ter, helix α2 and *C*-ter (Fig. 6a), the key components of ASP1's cavity ‘entrance’. Among these H-bonds, the H-bond pairs contributed by Val34-Trp116 and Asp35-Ile119 are vital for stabilizing the relative position of *C*-ter and α2. At pH 4.5, the carboxylic side-chain of protonated Asp35 can form an H-bond with oxygen atoms at carboxyl of Ile119. These two H-bonds appear to be complementary to each other (Fig. 6b) and present about 50% of the trajectory time. However, at pH 7.0, the distances between Asp35 and Ile119 are about 6 Å (Fig. 6b), thus these H-bonds can not form when Asp35 is in an unprotonated state at pH 7.0, until the moment that ligand starts leaving the cavity (e.g. about 40 ns in Fig. 6b). At the same time, the H-bond between Trp116 (N) and Val34 (O) occupies about 17% over the trajectory time at pH 4.5, in contrast to less than 1% at pH 7.0 (Table 1). The H-bond formed by Lys17 and Ile119 is another dynamic factor in stabilizing the cavity entrance of ASP1 (Fig. 6a). At pH 4.5 condition, the established H-bond occupies about 40% of simulation time while only 30% at pH 7.0 condition (Table 1).

It is important to note that the H-bonds contributed by Ile119 interplay with Lys17 and Asp35 to stabilize *C*-ter and *N*-ter (Figure S2.4 in **Supporting Information S2**). This interplay may help the domain swap of *N*-ter at pH neutral condition [31] or potential dimerization [9]. This hypothesis could be proved, since an H-bond between Trp4 and Val118 can be formed (At pH 4.5, this H-bond takes about 8% of simulation time while only near zero under the pH 7.0 condition), where a mutated ASP1 could only remain monomeric form [31]. Meanwhile, the H-bond between Tyr48 and Ile119, which is important in the crystal structure [7], is indeed found to be playing an active role during our dynamics simulation (12.17% occupation time at pH 4.5 vs. 2.92% at pH 7.0).

Through H-bond networks, for ASP1 at pH 4.5 condition, its *C*-ter is largely locked by H-bond formed by residues of Val118, Ile119 and Lys17, Asp35, Tyr48. H-bonds apparently constrain *C*-ter from folding into the binding pocket so as to keep the ligand bound in cavity. Nonetheless, once this H-bond network is broken at pH 7.0, particularly around the *C*-ter and α2 region, then the highly dynamic and flexible *C*-ter will open up the cavity and allow the release of the odorant ligands. This clearly solves the experimentally observed puzzle [7] on how pH affects the structures of ASP1. It demonstrates the pH-sensing ‘lock’/‘unlock’ mechanism proposed by Leal and coworkers [9].

## Conclusions

Comprehensive molecular simulations of OBP ASP1 in *apo* and *holo* states at different pH conditions were carried out. In *apo* state, *C*-ter will strike into the cavity and the global conformation undergoes large fluctuations. When the ASP1 and ligand complex were set at low or neutral pH conditions, we found that the ligand binding cavity formed dynamic ‘entrance’ geometry (by *C*-ter, *N*-ter, helix α2 and helices α4 and α5) and it responds to the changing pH condition. Interestingly the ionizable residues, which answer to pH change with protonation or unprotonation, do not directly interact with the ASP1 ligands; rather, the protonation affects the overall flexibility and fluctuation. There is clearly a process of dynamics propagation in ASP1 (and other OBPs) in response to pH stimuli. ASP1 is found to bind in favor of low pH conditions. H-bonds formed at the cavity entrance play an important role in regulating the ligand release, as indicated by ASP1 exposed from low pH to neutral pH conditions. The H-bond network carries on the dynamics change induced by varying the pH condition and passes on to the global change of flexibility and fluctuations of the OBP-ligand complex.

In summary, in OBPs/PBPs the ligand binding or releasing is very sensitive to pH conditions, and *C*-ter must cooperate with other key dynamic components for effective operation. Given there are different OBP families with characteristic *C*-ter, our finding can provide insightful molecular understanding of the mechanism and dynamics of OBPs, and further harness this understanding to biotechnology and agricultural applications.

## Materials and Methods

### The ASP1 structures and starting states

The molecular OBP structures were set up based on the crystal structures of bee antennal pheromone-binding protein ASP1 [32]. ASP1 typically has six tightly packed helices linked by short unstructured loops (Fig. 1a & 1b, with the odorant molecule of palmitic acid as shown in Fig. 1c). There are about 20 crystal structures of ASP1 in the states of native *apo* or complex with ligands. A *holo* form of crystal structure with the palmitic acid ligand (PDB code: 3BFH [7]) and an *apo* one (PDB code: 2H8V [7]) were used as our initial structures. All the crystal water molecules and other small molecules in the protein structures were removed before modelling. The missing residues (Asp3) at *N*-terminus of the *holo* structure were rebuilt in order to match the full length sequence of *apo* ASP1 structures. To assess reasonable ASP1 states/structures with or without odorant ligand against the benchmark, two other model ASP1 structures were created for molecular dynamics simulation. Based on PDB:3BFH and PDB:2H8V, one mimic structure is the complex with the ligand docking into the *apo* ASP1 structure (i.e. PDB:2H8V), and the other is an *apo* state with the ligand deleted from the *holo* state (i.e. PDB:3BFH). Consequently, total of four *apo* or *holo* starting systems were set up for simulations either at low or neutral pH conditions: These are ‘*apo*-2H8V’ and ‘*holo*-3BFH’, two states starting from ASP1 crystal structures plus ‘*holo*-2H8V’ and ‘*apo*-3BFH’ the mimic states, with all residues set at desirable protonated states (see **Supporting Information S1** and **Supporting Information S3**).

### Molecular docking and dynamics simulations

To create a ligand-bound state of ASP1, AutoDock 4.2 program [33] was used to dock palmitic acid into *apo* ASP1 structures (for more details please see **Supporting Information S1**). The lowest energy conformation of the complex structure, in which ligand had the similar conformation as the ligand in *holo* ASP1 structure, was selected as the initial structure for molecular dynamics (MD) simulation.

We performed all atom MD simulation on *apo* and *holo* states at different pH conditions to examine how pH affects the dynamics of ASP1 and its interactions with ligands. In order to capture the odorant releasing mechanism as per being influenced by pH, we focused our simulations on the *holo* states with varying pH conditions. All MD simulation systems were prepared and visualized with VMD [34].

MD simulations were performed on NAMD (version 2.8) [35] with the CHARMM27 force field [36], [37]. For each system, 200 ns MD production was performed at NPT ensemble, keeping the temperature at 300k, and the conformations were conserved every 0.1ps for subsequent analysis. For the *holo* states of ASP1, three replicates of each pH condition with random initial velocities were executed in order to explore more conformational space of ASP1 More molecular simulation details can be found in **Supporting Information S1**.

### Relative binding energy calculation

To quantitatively evaluate the perturbation of palmitic acid and ASP1, free energy perturbation (FEP) method can be used to calculate the binding energy difference of palmitic acid and ASP1 under different pH conditions. According the thermo- dynamics cycle (see Figure S2.3a in **Supporting Information S2**), the relative difference of binding energy could be measured as Eq. (1).
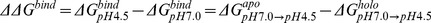
(1)Thus a relative negative/positive value of ΔΔ*G* ^bind^ shall indicate whether binding at pH 4.5 is more or less preferable than at pH 7.0. For details on the FEP binding energy calculation, please see **Supporting Information S1**.

### Dynamics analysis

The conformational changes with 0.1ps intervals were extracted from MD trajectories and analyzed using VMD and Wordom programs [38]. Backbone atoms root mean-square deviation (RMSD, versus the time) and root mean-square fluctuation (RMSF, versus the residues) were measured using Wordom. For hydrogen bond analysis, the distance and angle cutoff were set as 3.0 Å and 20 degree, respectively.

In this work, the center of selected residues was represented with the geometrical center of *C* ^α^ atom of each residue, as in our previous work [39]. Each residue in the selected region was represented by its *C* ^α^ atom and the atomic coordinates of the geometrical center of this region were calculated as follows:
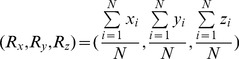
(2)Where *R*_x_, *R*_y_ and *R*_z_ are the coordinates of the center of the selected residues; *x*_i_, *y*_i_ and *z*_i_ are the trajectory of the *C^a^* atom in residue *i*; while *N* is the total number of selected residues.

To measure the compactness of the protein structure, the radius of gyration, *R*_g_, was calculated for each protein conformation by, 
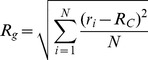
(3)where *N* represents the number of selected atoms. In our work, the backbone atoms of protein structure were chosen. *R*_C_ is the center of the protein structure as calculated using Eq. (2), and ***r***_i_ is the position of each backbone atom.

For MD simulations of the *holo* and *apo* states at different pH condition, the analysis were carried out by the average of three replicates. To clear the tendency of data, the cubic smoothing spline was fitted to the time evolution data using the smooth.spline function in *R* program. The secondary structure of each conformation was analyzed with DSSP program [30]. The sequences were aligned using PRALINE server [40] and multiple structural alignments of OBP structures were done with MultiProt program [41]. All the figures were prepared using PyMol and *R* program.

## Supporting Information

Movie S1 The full trajectories of 200 ns MD simulation of *holo* ASP1 under pH 4.5 and pH 7.0 conditions, respectively.
(MPG)

Movie S2 The full trajectories of 200 ns MD simulation of *holo* ASP1 under pH 4.5 and pH 7.0 conditions, respectively.
(MPG)

Supporting Information S1 Detailing the protonation, molecular dock, simulation procedures and calculation of binding energy by free energy perturbation (FEP) method.
(DOCX)

Supporting Information S2 Showing the fluctuations, disulfide bonds, H-bond, and thermodynamic cycles utilized in FEP calculations as per influenced by pH conditions, respectively.
(DOCX)

Supporting Information S3 The calculated p*K*_a_ values for ASP1 residues before protonation treatment.
(DOCX)
