# Supplementary material for: Operating Mechanism and Molecular Dynamics of Pheromone-Binding Protein ASP1 as Influenced by pH
Source: PLoS One. 2014 Oct 22;9(10):e110565. doi: 10.1371/journal.pone.0110565 (PMC4206424; doi:10.1371/journal.pone.0110565)
Supplement: Supporting Information S1 — Detailing the protonation, molecular dock, simulation procedures and calculation of binding energy by free energy perturbation (FEP) method. (DOCX) [file pone.0110565.s003.docx]

**Supplementary & Supporting Materials:**

‘Operating mechanism and molecular dynamics of pheromone-binding protein ASP1 as influenced by pH’ by Lei Han, Yong-Jun Zhang, Long Zhang, Xu Cui, Jinpu Yu, Ziding Zhang, and Ming S. Liu

**Supplementary Materials and Methods S1:**

**S1.1 Protonation of residues at designed pH conditions**

In the MD simulation forces, with changing physiological pH, the electrostatic interactions amongst the different regions of the protein (mainly the acidic residues) and vicinal water are mediated by the ionic strength, namely the pH conditions. For example, amino acids of the charged residues whose ionization state is highly sensitive to pH consistently have p*K*a shifted from their normal values. We take this protonation property to mimic the impact of changing pH conditions. Technically we chose the protonation state of each residues in the protein by validating an initial good guess with a standard p*K*a calculation (i.e. the residues are assigned charge states according to the set pH

values). Firstly we guess what their protonation is assumed to be, and eventually check them later with snapshots from the MD trajectory. This treatment of setting up pH conditions has been a stand practice as reported by hundreds on a wide range of protein systems.

The protonated state of ionizable residues were determined based on the p*K*_a_ values predicted by H++ server [1]. For one ionizable residue, if its p*K*_a_ value was greater than the set pH of the system, this residue was then protonated. Otherwise, the residue needed no protonation. To investigate the dynamic properties of ASP1 structure at different pH conditions, the ASP1-odorant systems were set to low pH 4.5 and neutral pH 7.0, respectively. For the single histidine (His) in ASP1 structure, it was protonated at both different pH conditions based on the prediction of H++ server (p*K*_a_ = 7.11). The detailed p*K*_a_ values of ionizable residues are listed in supplementary Table S1.

**S1.2 Molecular docking**

To create a ligand-bound state of ASP1, AutoDock 4.2 program [2] was used to dock palmitic acid into *apo* ASP1 structure. AutoDock Tools version 1.5.6 was used to prepare the materials for molecular docking and to analyze the docking results. The grid maps with a spacing of 0.375 Å were generated by AutoGrid. 50 runs were produced using Lamarckian Genetic Algorithm (LGA) algorithm [3], with 2.5 x 10^7^ energy evolution per run. All other parameters were set to default AutoDock values. The lowest energy conformation of complex, in which ligand had the similar conformation as the ligand in *holo* ASP1 structure, was selected as the initial structure for molecular dynamics simulation.

**S1.3 Molecular dynamics simulations**

We performed all atom molecular dynamics (MD) simulation on the *apo* and *holo* states of ASP1 at different pH conditions to examine how pH affect the dynamics and ASP1-ligand interactions. In order to capture the odorant releasing mechanism as per influenced by pH, our simulations were focused on the *holo* states with varying pH conditions. All molecular dynamics simulation systems were prepared and visualized with VMD [4]. The TIP3P model waters were used to solve the ASP or ASP-ligand complexes in a periodic boundary cubic box extending up to 10 Å from the protein to boundary in each direction [5]. Sodium and chloride ions, with concentration of 0.1M, were added to neutralize the net charge of the OBP and water systems.

MD simulations were performed on NAMD (version 2.8) [6] with the CHARMM27 force field [7,8]. Firstly, the systems were energy minimized for 5000 steps and then 100 ps of simulation was performed with solute restrained. Three similar processes were carried out with restraining heavy atoms of protein, ligand and backbone of protein respectively. After that, the temperature of systems were gradually heated to 300K during 30ps Langevin dynamics using a time step of 1fs following 5ns system NVT equilibration without any restraint. For each system, 200ns MD production runs were performed at NPT ensemble keeping the temperature at 300k and the conformations were conserved every 0.1ps for subsequent analysis. For MD production running, the temperature and pressure of systems were controlled using Langevin dynamics with a damping coefficient of 1 ps^-1^ and Nose’-Hoover methods, respectively. Van der Waals force was truncated at 12 Å and particle Mesh-Ewald (PME) summation scheme was used to handle the long range electrostatic interaction [9]. For explore more comprehensive conformational space of ASP1 at different pH conditions, three replicates of MD were produced for each pH condition (pH4.5 and pH7.0) and subsequent dynamics analysis (such as RMSD, RMSF, distance between residues or component of ASP1) were conducted by averaging the triplicates.

**S1.4 Free energy perturbation calculation**

The relative binding energy for the ASP1-ligand complexes was calculated using free energy perturbation (FEP) method [[10](#_ENREF_10)], where in FEP calculation the thermodynamics cycle was followed as in Fig. S3.a. In our FEP calculation process, dual topology approach was used [[11](#_ENREF_11)], with the initial state set at (λ = 0, pH4.5 condition and the final state set at (λ = 1, pH7.0) respectively. In MD simulations with the system transiting from the initial state to the final state, the relative binding energy difference was calculated as [11],

$\Delta\Delta G_{\lambda=0\to\lambda=1}=-\frac{1}{\beta}\sum_{\lambda=0}^{\lambda=1} \ln\left\langle\exp(-\beta(H_{(\lambda+\delta\lambda)}-H_{(\lambda)}))\right\rangle_{\lambda}$ (s1)

Here ***β***=$\frac{1}{k_{B}T}$, ***k***_B_ is the Boltzmann constant, ***T*** is the temperature and ***H*** is the total energy of system. λ = 0 and λ = 1 represented the *holo*-ASP1 in pH4.5 and pH7.0 states, respectively. In our FEP process, the standard stepping δλ was set as 0.05. However, at the start and close to the final states, the interval sequence of λ, i.e. δλ, was set as {0, 0.00001, 0.0001, 0.001, 0.01, 0.03, 0.05, 0.08, 0.1} and {0.9, 0.92 ,0.95, 0.97, 0.99, 0.999, 0.9999, 0.99999, 1}, which in total we had 32 intermediate λ states (i.e. total of 16 intermediate λ states around the start and final states and other 16 intermediate states were assigned to process λ from 0.1 to 0.9). At each λ state, 200ps simulation was run to equilibrate the ASP1-ligand system and then 500ps simulation was produced to collect the energetics data. Other parameters for FEP calculation were set the same as the standard MD simulation.

**Supplementary references:**

1. Gordon JC, Myers JB, Folta T, Shoja V, Heath LS, et al. (2005) H++: a server for estimating pKas and adding missing hydrogens to macromolecules. Nucleic Acids Res 33: W368-W371.

2. Morris GM, Huey R, Lindstrom W, Sanner MF, Belew RK, et al. (2009) AutoDock4 and AutoDockTools4: Automated docking with selective receptor flexibility. J Comput Chem 30: 2785-2791.

3. Morris GM, Goodsell DS, Halliday RS, Huey R, Hart WE, et al. (1998) Automated docking using a Lamarckian genetic algorithm and an empirical binding free energy function. J Comput Chem 19: 1639-1662.

4. Humphrey W, Dalke A, Schulten K (1996) VMD: Visual molecular dynamics. J Mol Graph 14: 33-38.

5. Jorgensen WL, Chandrasekhar J, Madura JD, Impey RW, Klein ML (1983) Comparison of simple potential functions for simulating liquid water. J Chem Phys 79: 926-935.

6. Phillips JC, Braun R, Wang W, Gumbart J, Tajkhorshid E, et al. (2005) Scalable molecular dynamics with NAMD. J Comput Chem 26: 1781-1802.

7. MacKerell AD, Bashford D, Bellott, Dunbrack RL, Evanseck JD, et al. (1998) All-Atom Empirical Potential for Molecular Modeling and Dynamics Studies of Proteins†. J Phys Chem B 102: 3586-3616.

8. MacKerell AD, Banavali N, Foloppe N (2000) Development and current status of the CHARMM force field for nucleic acids. Biopolymers 56: 257-265.

9. Darden T, York D, Pedersen L (1993) Particle mesh Ewald: An Nlog(N) method for Ewald sums in large systems. J Chem Phys 98: 10089-10092.

10. Kollman P (1993) Free energy calculations: Applications to chemical and biochemical phenomena. Chemical Reviews 93: 2395-2417.

11. Pearlman DA (1994) A Comparison of Alternative Approaches to Free Energy Calculations. The Journal of Physical Chemistry 98: 1487-1493.
