# Supplementary material for: Operating Mechanism and Molecular Dynamics of Pheromone-Binding Protein ASP1 as Influenced by pH
Source: PLoS One. 2014 Oct 22;9(10):e110565. doi: 10.1371/journal.pone.0110565 (PMC4206424; doi:10.1371/journal.pone.0110565)

**Supplementary Figures S2:**

‘Operating mechanism and molecular dynamics of pheromone-binding protein ASP1 as influenced by pH’ by Lei Han, Yong-Jun Zhang, Long Zhang, Xu Cui, Jinpu Yu, Ziding Zhang, and Ming S. Liu

**Figure S2.1** Backbone’s RMSD (a) and RMSF (c) of the *apo* and *holo* states of 2H8V and 3BFH simulated at pH4.5 condition. RMSD (b) and RMSF (d) of *holo*-3BFH simulated at pH4.5 and pH7.0 conditions, respectively. In (a) and (c), the dotted lines show the RMSDs from the initial structure for each repeated trajectory (e.g. 6 RMSD plots for the *holo* state).

^
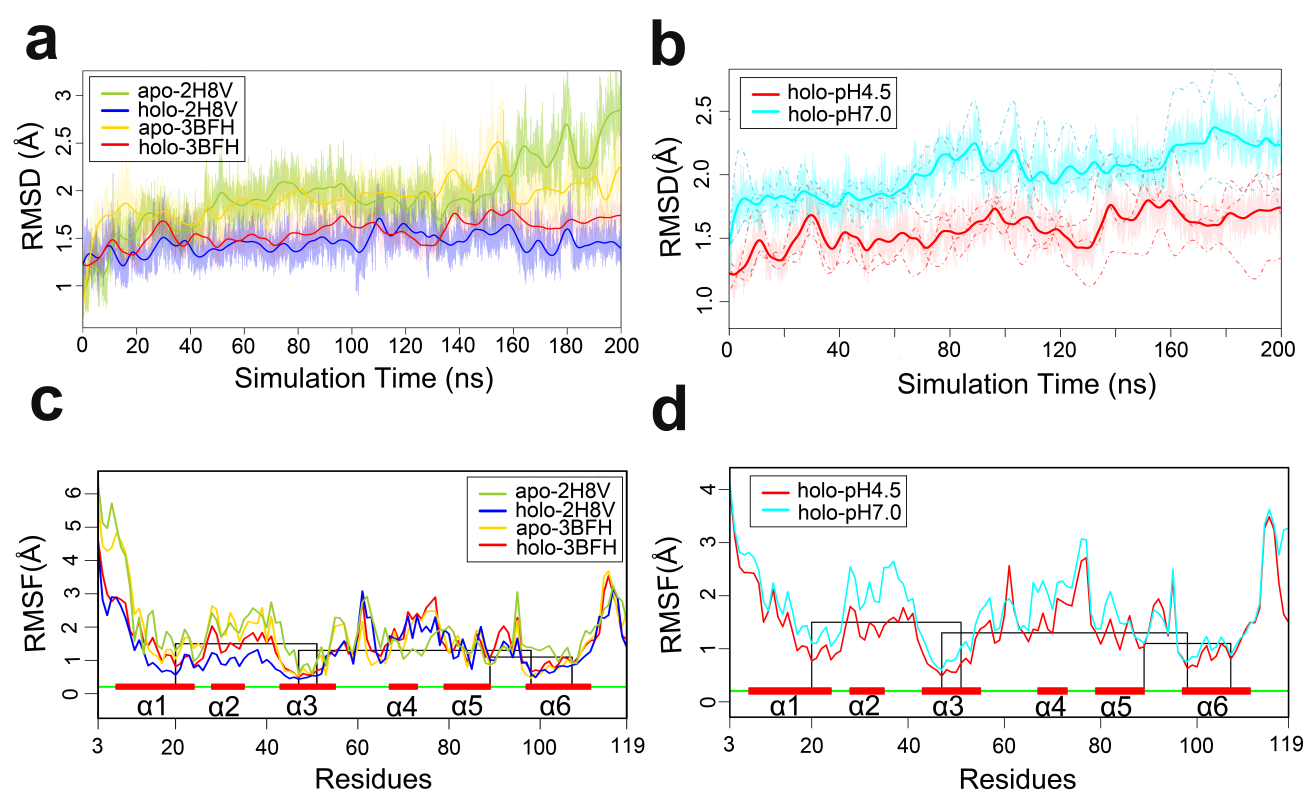
^

**Figure S2.2** The disulfide bond plane in ASP1. (a) The centers of these three disulfide bond form the disulfide bond plane. The cysteines are highlighted with ball-and-stick model. (b) The disulfide plane fluctuation with time evolution. The crystal structure (PDB code: 3BFH) was selected as reference and the planar dihedral angle shows the variation of each conformation from the reference state.

^
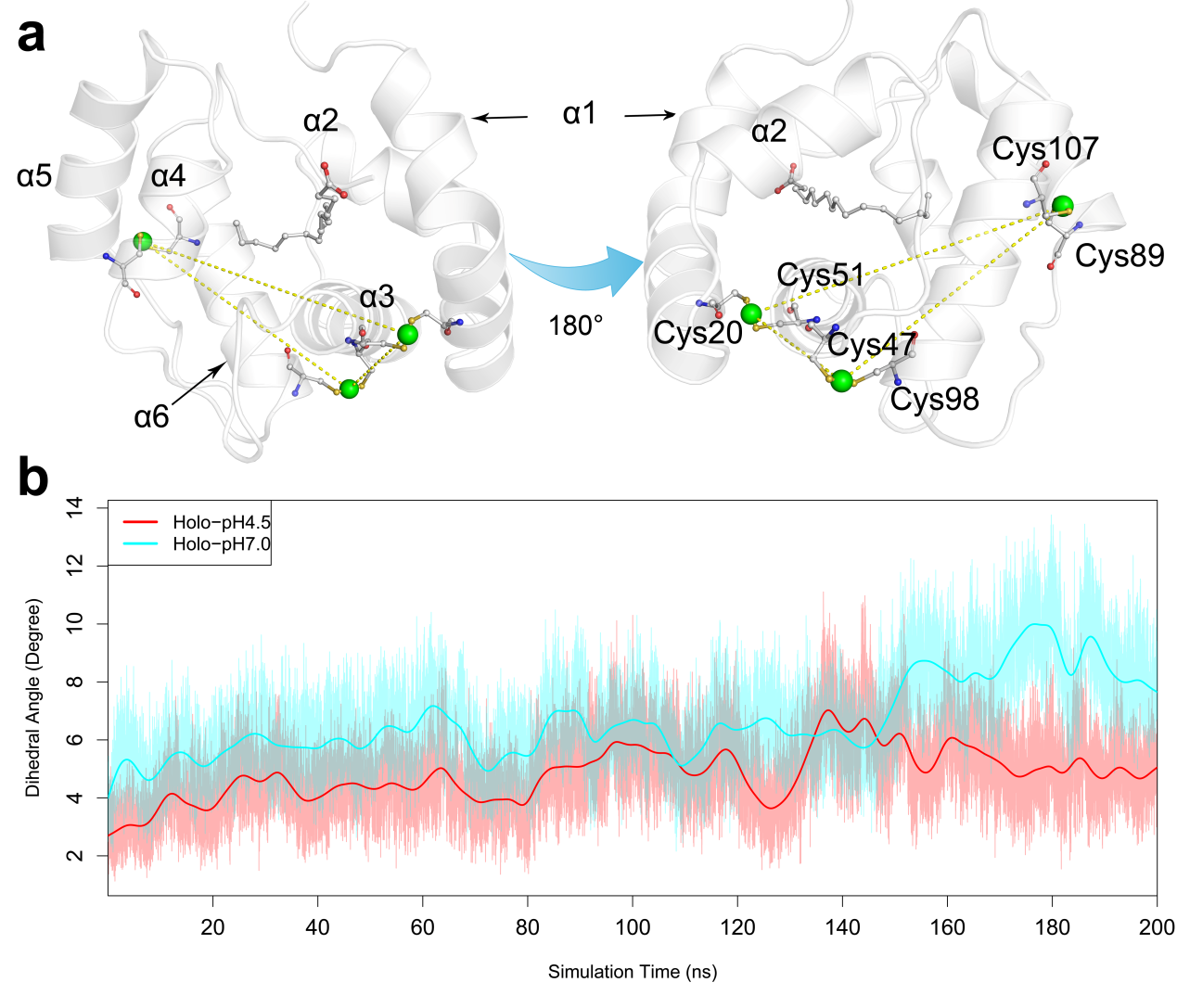
^

**Figure S2.3** Free energy perturbation (FEP) calculation of ASP1. (a) Thermodynamic cycles utilized in the FEP calculations; (b) free energy variation as ASP1 transits from *apo*- at pH 4.5 to *holo*- at pH7.0.


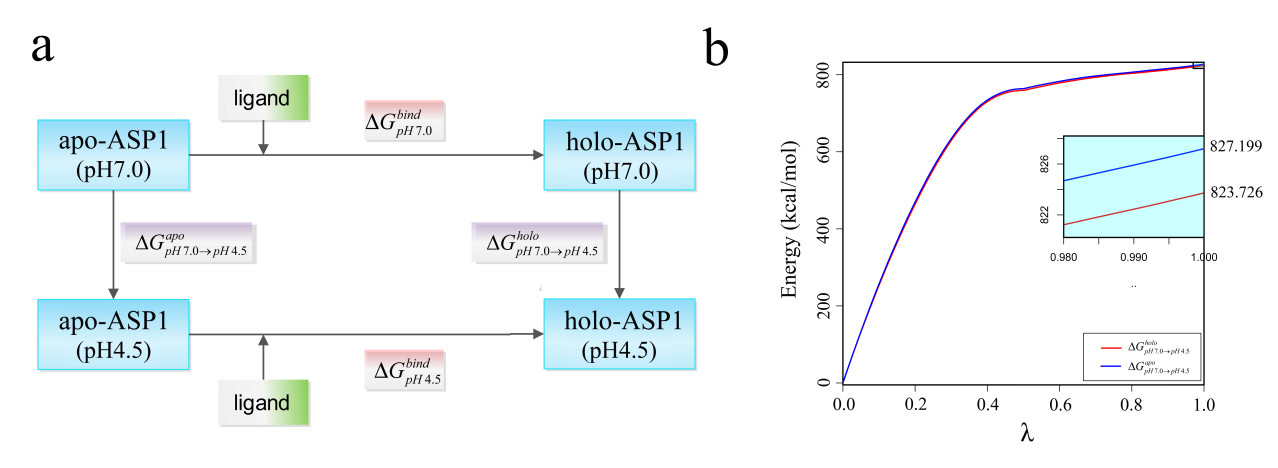


**Figure S2.4** The bond length and occupation time of potential H-bonds formed between Ile119 and Lys17 or Asp35 at pH4.5 and pH 7.0 conditions, respetively. The trajectory beyond about 80ns indicates a closure motion of *C*-terminal under low pH condition, mainly driven by residue Asp 35.


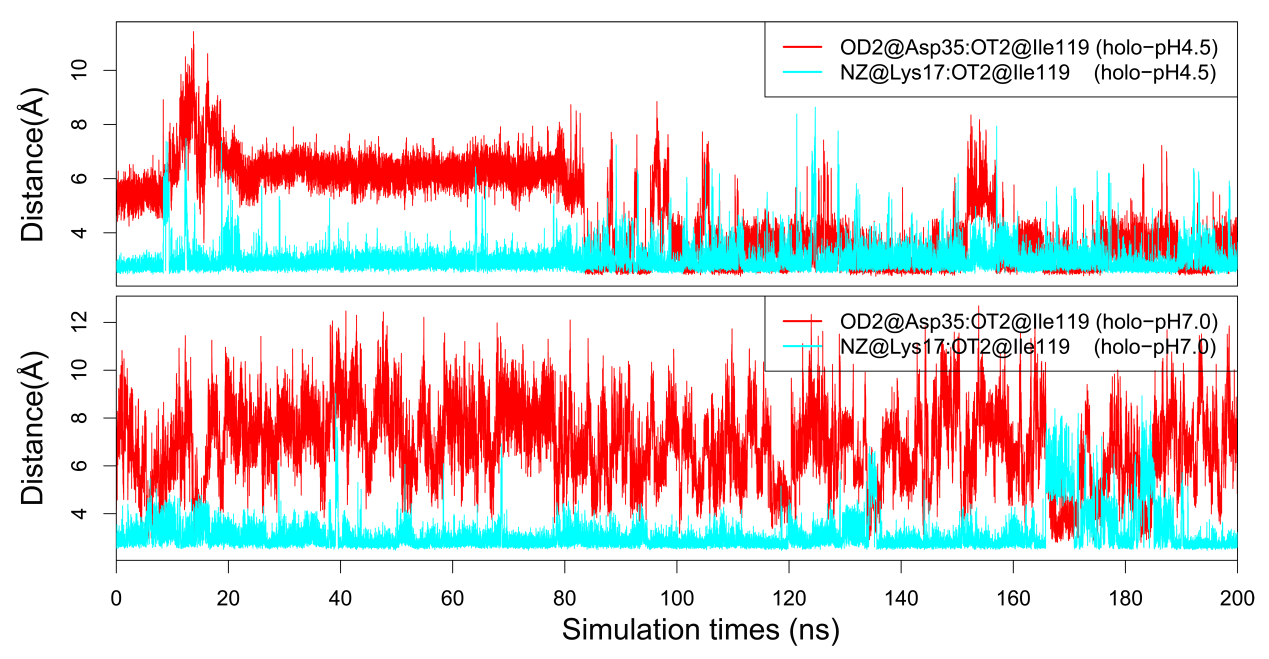

Supplement: Supporting Information S2 — Showing the fluctuations, disulfide bonds, H-bond, and thermodynamic cycles utilized in FEP calculations as per influenced by pH conditions, respectively. (DOCX) [file pone.0110565.s004.docx]
