# Supplementary material for: Operating Mechanism and Molecular Dynamics of Pheromone-Binding Protein ASP1 as Influenced by pH
Source: PLoS One. 2014 Oct 22;9(10):e110565. doi: 10.1371/journal.pone.0110565 (PMC4206424; doi:10.1371/journal.pone.0110565)
Supplement: Supporting Information S3 — The calculated pK a values for ASP1 residues before protonation treatment. (DOCX) [file pone.0110565.s005.docx]

**Supplementary Table S3:**

The p*K*_a_ value of ionizable residues for PDB 2H8V and 3BFH. The p*K*_a_ value was calculated using the H++ program (<http://biophysics.cs.vt.edu/H>++). In this table, NTASP represents the amino-group of N-terminus and CTILE meant the carboxyl of the C-terminus.

| Residue | 2H8V | 3BFH |
| --- | --- | --- |
| NTASP-3 | 8.829 | 8.944 |
| aspNT-1 | 4.399 | 2.870 |
| GLU-8 | 4.893 | 4.810 |
| ASP-11 | 4.260 | 4.229 |
| GLU-15 | 4.683 | 4.730 |
| ASP-16 | 3.715 | 3.577 |
| LYS-17 | 15.587 | 16.585 |
| ARG-19 | 16.134 | 15.474 |
| GLU-23 | 3.425 | 3.183 |
| HIS-24 | 7.097 | 7.172 |
| ASP-32 | 3.640 | 3.775 |
| ASP-33 | 4.266 | 4.850 |
| ASP-35 | 8.752 | 10.561 |
| LYS-36 | 11.675 | 11.578 |
| GLU-42 | 4.155 | 4.170 |
| TYR-48 | 30.140 | 29.843 |
| TYR-50 | 17.689 | 18.493 |
| GLU-54 | 5.615 | 5.206 |
| ASP-60 | 2.064 | 1.553 |
| ASP-61 | 5.014 | 5.050 |
| GLU-62 | 4.408 | 4.608 |
| ASP-66 | 5.229 | 5.236 |
| GLU-67 | 5.952 | 5.414 |
| ASP-68 | 4.188 | 4.152 |
| ASP-76 | 4.386 | 4.460 |
| GLU-80 | 4.295 | 4.389 |
| ARG-81 | 12.897 | 13.217 |
| LYS-88 | 11.775 | 11.466 |
| ASP-96 | 2.285 | 2.773 |
| LYS-100 | 11.845 | 12.274 |
| TYR-102 | 10.168 | 11.874 |
| LYS-106 | 14.171 | 11.349 |
| GLU-110 | 4.904 | 4.010 |
| ASP-114 | 4.662 | 1.719 |
| CTILE-119 | 1.225 | 1.928 |
